# THE UTILITY AND POTENTIALS OF CLAIMS-BASED FRAILTY INDEX IN CLINICAL CARE AND RESEARCH: NEW DEVELOPMENTS IN 2024

**DOI:** 10.1093/geroni/igae098.1259

**Published:** 2024-12-31

**Authors:** Dae Hyun Kim

**Affiliations:** Chair

## Abstract

The development of a claims-based frailty index (CFI) for Medicare claims (Kim et al., Journals of Gerontology: Medical Sciences, 2018) has empowered researchers to integrate frailty into pharmacoepidemiology and health services research. This symposium aims to overview the CFI’s development and validation process and spotlight its recent applications in studies utilizing Medicare claims and electronic health records (EHR). The CFI, estimating a deficit accumulation frailty index (range: 0 to 1, with higher values indicating greater frailty) based on the previous year’s claims, demonstrates 60% sensitivity and 86% specificity for a deficit accumulation frailty index ≥0.25, and 62% sensitivity and 78% specificity for the Fried Frailty Phenotype. Additionally, a crosswalk between CFI and clinical frailty measures is available, aiding the translation of research findings into clinical practice. Featured presentations will explore the CFI’s application to EHR data, its role in assessing dementia severity, its contribution to the CMS Hierarchical Condition Category model in predicting healthcare costs, its predictive value for outcomes following post-acute care in skilled nursing facilities, and its association with opioid discontinuation rates in older adults hospitalized with fractures. These examples will highlight the benefits and challenges of using CFI in research.

